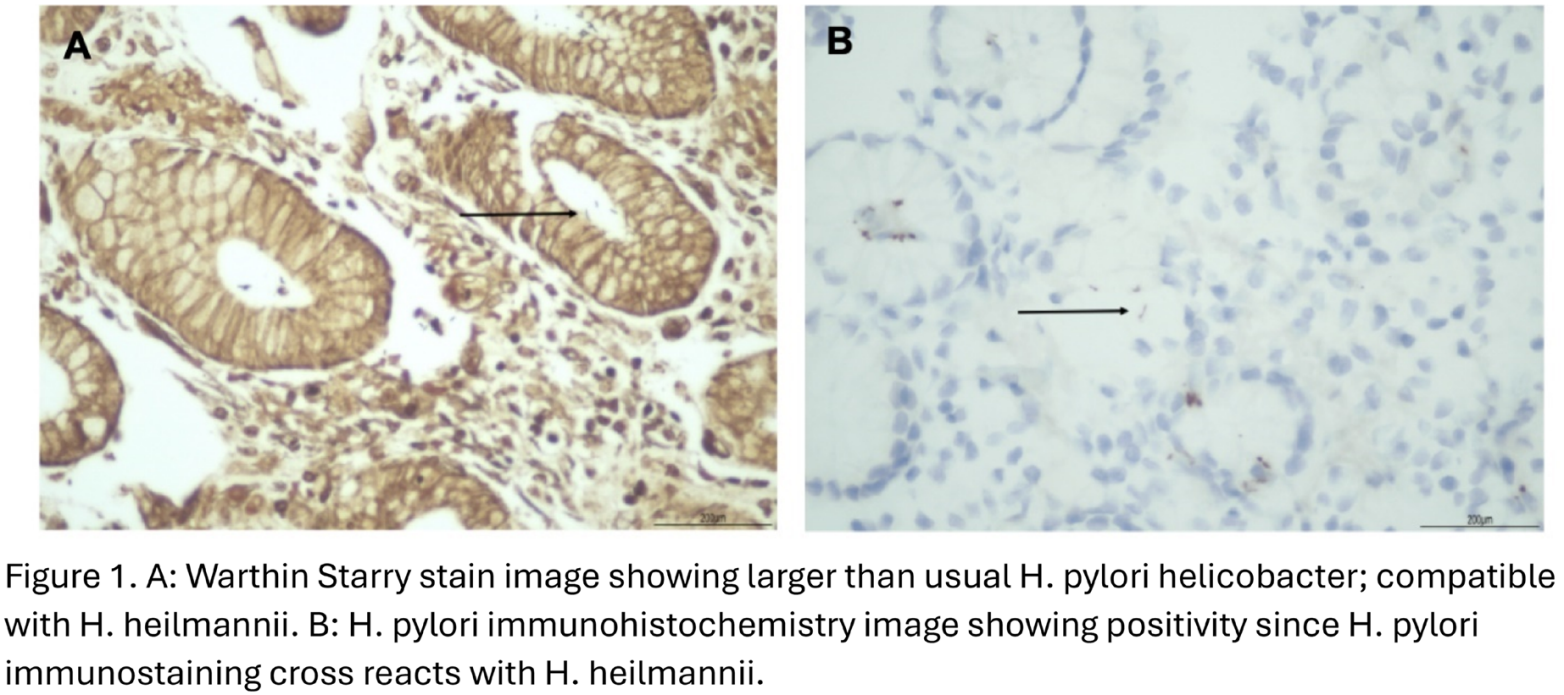# Poster Session I - A136 BEYOND H. PYLORI: A RARE CASE OF REFRACTORY HELICOBACTER HEILMANNII GASTRITIS LINKED TO IRON DEFICIENCY ANEMIA

**DOI:** 10.1093/jcag/gwaf042.136

**Published:** 2026-02-13

**Authors:** S Niki, D D’Urbano, F Jowhari

**Affiliations:** The University of British Columbia Faculty of Medicine, Vancouver, BC, Canada; Department of Pathology and Laboratory Medicine, University of British Columbia Okanagan, Kelowna, BC, Canada; Division of Gastroenterology, Department of Medicine, University of British Columbia Okanagan, Kelowna, BC, Canada

## Abstract

**Background:**

Iron deficiency anemia (IDA) remains a leading cause of global morbidity. While Helicobacter pylori–associated gastritis is a recognized cause of chronic blood loss, Helicobacter heilmannii is a rare, non-H. pylori gastric Helicobacter (NHPH) detected in < 1% of gastric biopsies in western populations. H. heilmannii has been linked to gastritis, ulcers, and gastric MALT lymphoma. Evidence linking H. heilmannii to IDA is limited, and standardized diagnostic or eradication protocols are undefined.

**Aims:**

To describe a case of H. heilmannii gastritis identified during investigation of unexplained IDA and to outline diagnostic, therapeutic, and eradication challenges, including failure of standard H. pylori regimens.

**Methods:**

A man in his 60s underwent evaluation for asymptomatic iron deficiency anemia (Ferritin 5.1 µg/L). Celiac serology and Fecal Immunochemical testing were negative, and Colonoscopy was unremarkable. Gastroscopy revealed distal esophagitis while gastric biopsies showed mild chronic gastritis with large spiral organisms seen on Warthin–Starry staining consistent with H. heilmannii. Immunohistochemistry was positive for H. pylori likely due to cross-reactivity. The patient received sequential eradication regimens with a 14-day bismuth-based quadruple therapy, 14-day levofloxacin-based triple therapy, and salvage rifabutin-amoxicillin-PPI therapy after Infectious Diseases consultation. Iron stores were replenished with supplementation. Serial urea breath tests were used for test-of-cure (TOC).

**Results:**

Ferritin and hemoglobin failed to normalize with oral iron (ferritin 5.8 µg/L; Hb 111 g/L) but corrected following six intravenous infusions (ferritin 119.8 µg/L; Hb 136 g/L). H. heilmannii persisted on two successive urea breath tests after both first and second-line therapies. Salvage rifabutin-based therapy was initiated and repeat eradication testing is pending. The patient has remained asymptomatic throughout.

**Conclusions:**

This case highlights H. heilmannii as an uncommon cause of gastritis discovered during IDA evaluation. Correction of IDA despite persistent infection suggests a coincidental relationship rather than causality. Failure of standard H. pylori regimens underscores that H. heilmannii may demonstrate distinct antibiotic susceptibility. Urea breath testing, while commonly used, remains an imperfect surrogate for TOC due to non-specific urease activity. Stool antigen testing also lacks validation for NHPH. Rifabutin-based salvage therapy may represent a viable approach for refractory H. heilmannii. Further studies are needed to define standardized diagnostic, therapeutic, and TOC strategies for NHPH infections.

**Funding Agencies:**

None